# 

**Published:** 1908-06

**Authors:** 


					CONTENTS.
Page
's Opso
nic Treatment Useful in Phthisis?
cy John M. H. Munro, D.Sc. Lond.
97
alinette's Ophthalmic Reaction in Tuberculosis.
ey J. M. Fortescue-Brickdale, M.A., M.D. Oxon.
0rne Notes on the Treatment of Haemoptysis in Pulmonary
Tuberculosis.
By Newman Neild, M.B. Vict., M.R.C.P....
124
*>om
Clinical Points in the Early Diagnosis of Pulmonary
Tuberculosis.
By C. Muthu, M.D.
*31
I ase of Filariasis with Abscess.
By A. L. Flemming
139
Lach
rymal Obstruction: its Results, Dangers and Treatment.
"y Charles Goulden, M.A., M.B., M.C. Cantab., F.R.C.S. Eng.
143
pr0
gress of the Medical Sciences
Medicine.
G. Parker, M.D., M.A.Cantab.
152
Su
rgery.
E. H. E. Stack, B.A., M.B., B.C.Cantab.
157
P) Perinatology.
J. A. Nixon, B.A., M.B. Cantab.
i6o
?e*iews of Books
104
^'torial Notes
173
Not
>, tes on Preparations for the Sick
170
?. e Library of the Bristol Medico-Chirurgical Society   183
"eet
lr|gs of Societies
186
?c&l Medical Notes
i go

				

